# Distribution of the Phenotypic Effects of Random Homologous Recombination between Two Virus Species

**DOI:** 10.1371/journal.ppat.1002028

**Published:** 2011-05-05

**Authors:** Florence Vuillaume, Gaël Thébaud, Cica Urbino, Nadège Forfert, Martine Granier, Rémy Froissart, Stéphane Blanc, Michel Peterschmitt

**Affiliations:** 1 CIRAD, INRA, CNRS – Unité mixte de recherche Biologie & génétique des interactions plante-parasite (BGPI), Montpellier, France; 2 Laboratoire Maladies Infectieuses & Vecteurs: Écologie, Génétique, Évolution & Contrôle (MIVEGEC), CNRS-IRD-Université de Montpellier I, Agropolis, Montpellier, France; Cornell University, United States of America

## Abstract

Recombination has an evident impact on virus evolution and emergence of new pathotypes, and has generated an immense literature. However, the distribution of phenotypic effects caused by genome-wide random homologous recombination has never been formally investigated. Previous data on the subject have promoted the implicit view that most viral recombinant genomes are likely to be deleterious or lethal if the nucleotide identity of parental sequences is below 90%. We decided to challenge this view by creating a bank of near-random recombinants between two viral species of the genus *Begomovirus* (Family *Geminiviridae*) exhibiting 82% nucleotide identity, and by testing infectivity and *in planta* accumulation of recombinant clones randomly extracted from this bank. The bank was created by DNA-shuffling—a technology initially applied to the random shuffling of individual genes, and here implemented for the first time to shuffle full-length viral genomes. Together with our previously described system allowing the direct cloning of full-length infectious geminivirus genomes, it provided a unique opportunity to generate hundreds of “mosaic” virus genomes, directly testable for infectivity. A subset of 47 randomly chosen recombinants was sequenced, individually inoculated into tomato plants, and compared with the parental viruses. Surprisingly, our results showed that all recombinants were infectious and accumulated at levels comparable or intermediate to that of the parental clones. This indicates that, in our experimental system, despite the fact that the parental genomes differ by nearly 20%, lethal and/or large deleterious effects of recombination are very rare, in striking contrast to the common view that has emerged from previous studies published on other viruses.

## Author Summary

Recombination creates new genome combinations by joining genome fragments of distinct “parental” origin. This phenomenon, frequent in viral populations, combines mutations originally present on distinct parental genomes, increasing genetic diversity and creating “offspring” with altered biological properties. Consistently, recombination is often associated with the emergence of economically important viruses, with modified host range and virulence. In fact, recombination events can be lethal, deleterious or beneficial, but the respective frequency of these phenotypic effects is unknown and unpredictable. A generally accepted view, which we formally challenge in the present paper, is that most viral recombination events are deleterious or lethal when the parental sequences diverge by more than 10%. However, at present, no dedicated data set supports this supposition. We generated hundreds of “mosaic” genomes randomly from two plant virus species diverging by 18%, and tested a subset of 47 of these recombinants for viability and within-host accumulation. Surprisingly, all were viable, replicated, and accumulated at a pace comparable to that of the parents. Our results are in striking contrast to the current view, and show that viral recombination can have little phenotypic effect, at least in some cases, even when the parental sequences diverge by far more than 10%.

## Introduction

Genetic variation is generated primarily by inaccurate replication of genomes due to mutation and recombination. This is particularly true for viruses, which are characterized by high mutation rates [1], [2] leading to populations appearing rapidly as mutant swarms, and in which recombination can generate theoretically countless combinations of available mutations [2]. Remarkably, for some virus species, the intrinsic recombination rate calculated per nucleotide position and per replication cycle can approach, or even exceed, the estimated point mutation rate [3], [4], [5]. One major difference in the creation of new genotypes via mutation versus recombination lies in the fact that mutations are introduced individually, whereas recombination often introduces a group of mutations as a block in a new genomic context. Recombination can either disrupt existing interactions between mutations, or promote new interactions between a group of co-introduced mutations and the other part of the “recipient” genome. Another remarkable difference is that, unlike spontaneous mutations, those introduced by recombination might have been under selection pressure in their previous context. For these reasons, distribution of the phenotypic effects of mutation events might be very different from that of recombination events, as suggested by a remarkable study comparing the impact of these two phenomena on the functionality of an enzyme [6].

The distribution of the phenotypic effects of recombination in viruses has gone almost undocumented, in terms of both theoretical prediction and empirical investigation. Whereas experimental data on random point mutations revealed these to be mostly deleterious or lethal in a rhabdovirus infecting animals [7] as well as in a potyvirus infecting plants [8], to the best of our knowledge, experiments formally investigating the distribution of the phenotypic effects of random genome-wide recombination have never been reported. Previous experiments investigating the phenotype of small sets of recombinants did not address this question directly, and thus provided only incomplete information on the precise point investigated here. In these previous studies, the artificially-created recombinants were derived mostly from the exchange of specific, previously delimited, regions or genes suspected to be involved in known biological traits of the virus (e.g. [9], [10], [11]). Because such recombinants were usually less fit than the parental genomes [9], [10], [11], [12], particularly when their genetic identity was below 90% [10], it was suggested that, as with mutations, most recombination events create deleterious or even lethal genomic combinations. The fact that only a few specific recombinant types, rather than a wide range of random recombinants, are detected in natural viral populations [13], [14], [15], [16] seems to support this assertion, suggesting that only rare genomic combinations are fit enough to invade, or at least be maintained at a detectable frequency within the viral population.

Consequently, the view that recombination events between two viral genomes diverging by more than 10% are mostly deleterious or lethal has become widely accepted [12], [15], [17], [18], [19], despite the lack of direct and specific experimental support. In fact, as in equivalent studies on mutation [7], [8], such empirical support can be provided only by examining the phenotype of a large number of recombinants generated by a random process between two viral species. To date, the only examples in the literature where recombination was suggested to be random, according to recombination patterns determined in offspring genomes at early stages of co-infection (i.e. under minimum selection pressure), are those of a coronavirus infecting mice [20] and *Cauliflower mosaic virus* infecting turnip [3]. Unfortunately, neither of these studies allowed investigation of the biological properties of the recombinants, and no traits participating in viral fitness could be evaluated.

Here, using a huge bank of viral recombinants generated randomly *in vitro*, and with a cloning system enabling straightforward biological testing, we challenge the general assumption described above. Because the numerous recombinants tested in this study were generated in the absence of any selection pressure, our results provide information on all recombinant types, regardless of whether or not they resemble those found in natural populations.

We selected model viruses whose genomes are small enough to be shuffled throughout their entire length using a random gene shuffling technique. The selected parental viruses were from the family *Geminiviridae*, genus *Begomovirus*, and their genome consists of a single circular ssDNA of less than 3kb. This family was also selected because (i) a large number of recombinants have been isolated in the field [21], [22], (ii) some of these recombinants were found to be associated with new epidemic outbreaks [23], [24], [25], [26], and (iii) analysis of a large number of viral sequences accumulated in the databases has revealed recombination hot spots and cold spots distributed along the genome [18]. Most importantly, the selected parental genomes exhibited around 80% nucleotide identity, significantly below the virtual “90% threshold” for which maladapted recombinants have been frequently reported, discussed, and/or speculated on [10], [15], [16], [21].

We generated a bank of hundreds of shuffled recombinants, harbouring between 2 and 6 exchanged fragments of a size ranging from 1 to ≈2700 nucleotides (nt). A manageable subset of 47 recombinants was fully sequenced and tested individually for infectivity and accumulation *in planta*. Taking viral accumulation as a proxy for fitness, we show here that all 47 recombinants infected their host plant and accumulated in systemically colonized leaves at a level equal or intermediate to that of the parents. These results indicate that, at least for some virus species, lethality and large deleterious effects are surprisingly rare phenotypic outcomes of recombination.

## Results

### The bank of recombinant genomes

**Figure 1:**
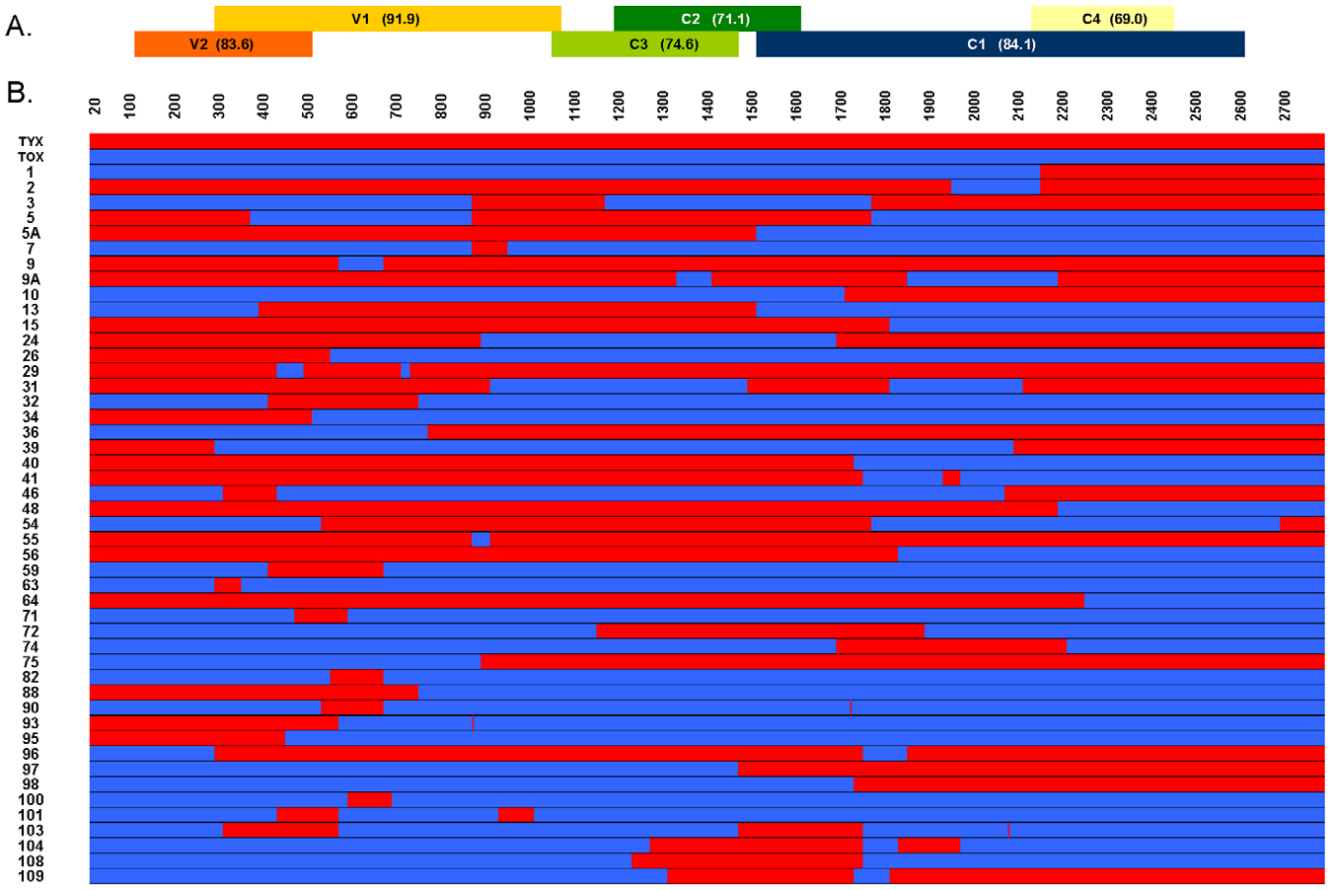
Schematic representation of 47 recombinant viral genomes generated by L-DNA-shuffling with the parental genomes of Tomato yellow leaf curl virus (Tyx) and Tomato leaf curl Mayotte virus (Tox). For convenience, each circular genome is presented in linear form beginning with the cloning site *Xho*I. (A) The 6 ORFs encoded by both parental genomes are presented above the aligned genomes: V2 [movement protein (MP)], V1 [coat protein (CP)], C3 [replication enhancer protein (Ren)], C2 [transcription associated protein (TrAP)], C1 [replication associated protein (Rep)], and C4 (symptom determinant implicated in cell cycle control). The percentage of amino acid identities between Tyx and Tox is indicated within parentheses for each ORF. (B) Recombinant genomes are represented in red for Tyx-derived fragments and in blue for Tox-derived fragments. Nucleotide positions are indicated above the aligned genomes.

The parental genomes, designated Tyx and Tox (2791 and 2765 nt in size, respectively) exhibit 82% overall nucleotide identity, thus the two sequences differ at about 500 nucleotide positions distributed throughout the genome (Figure S1A). As for all monopartite begomoviruses, the parental genomes encode six open reading frames (ORF), which exhibit different degrees of amino acid identity when comparing Tyx and Tox (Figure 1A).

Several hundred Tyx/Tox recombinant genomes were generated using L-DNA-shuffling technology as described in Materials and Methods. A total of 47 randomly selected shuffled genomes were fully sequenced (Figure 1B) and analysed for infectivity and accumulation *in planta*. Alignment with the parental sequences revealed that, among the 47 recombinants sequenced (representing a total of 130,478 nt), only 1 nt position in one genome (recombinant “2”) differed from both parents. None of the other recombinants encoded a non-parental nucleotide or amino acid. Each individual recombinant harboured between two and six breakpoints, corresponding to detectable exchange of 2 to 6 genomic segments.

Overall, we detected a total of 54 distinct breakpoints spread along the 47 recombinant genomes (Figure 1B and Figure S1B). The majority (29) of these breakpoints were found only once, but several (18) were found in more than one recombinant (Figure S1B). It is important to note that breakpoints can be located only roughly within a region separating two adjacent nucleotide positions where Tyx and Tox differ; thus, similar breakpoints identified in several recombinants as shown in Figure S1B are not necessarily identical. The size of the fragments generated by L-DNA-shuffling ranged from a few nucleotides to ≈2450 nt (Figure S2); the exchange of some short fragments resulted in a single nucleotide change. The number of shuffled fragments in the different size classes was rather homogeneous, although fragments with a size below 1100 nt (and especially below 100 nt) were more frequent. Only one breakpoint was detected in the intergenic region. All other breakpoints were located within genes, which is interesting for further phenotypic analysis: breakpoints were generated in all ORFs and 44 of the 47 recombinant genomes encode at least one hybrid protein. Finally, three recombinant genomes were modified only at the nucleotide level (synonymous changes): recombinants “7” and “10” encode 100% Tox proteins whereas recombinant “55” encodes 100% Tyx proteins. Overall, the bank of recombinants produced appears highly diverse, covering a wide range of possibilities in the number of exchanged DNA fragments per genome, their size and position. By picking clones randomly from this collection for further characterization (as described below) we are getting as close as possible to an analysis of the phenotypic effects of random recombination.

### Infectivity of the recombinant viruses

**Figure 2:**
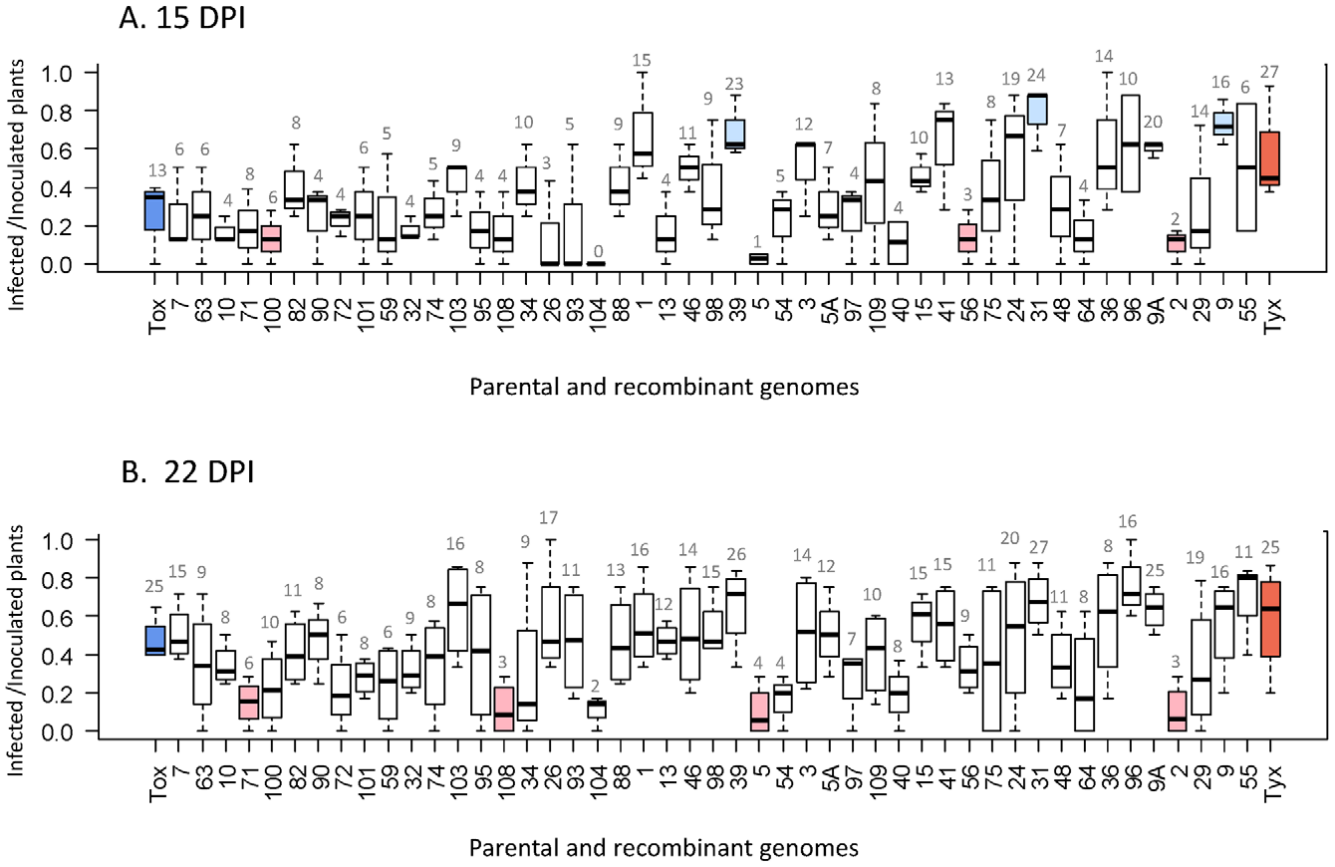
Infectivity of the 47 recombinants and their 2 parental genomes. Infectivity was determined on plant samples collected at 15 (A) and 22 days (B) post-inoculation. Infectivity was defined as the proportion of infected plants (virus detected in systemic leaves) of the total number of virus-inoculated plants. Within the boxes, the horizontal line indicates the median value (50% quantile), the box itself delimits the 25% and 75% quantiles, and the dashed lines represent the normal range of the values. The numbers on the top line indicate the number of infected plants for each viral clone. The blue box at the left end corresponds to the parental genome Tox and the red box at the right end to the parental genome Tyx. The recombinant genomes are ordered from left to right by increasing nucleotide identity with Tyx genome. White boxes correspond to recombinants that are not significantly different from either parent, light red boxes to recombinants that are significantly less infectious than Tyx, and light blue boxes to recombinants that are significantly more infectious than Tox.

Each of the 47 recombinant clones, as well as the two parental viral clones, were agro-inoculated individually onto tomato plants in four independent repeated tests. Infectivity was defined at a given date as the proportion of infected plants (virus detectable in systemically infected leaves) of the total number of virus-inoculated plants. Unexpectedly, all 47 recombinants were detected in systemically infected leaves in at least some of the inoculated test plants, indicating that none of them was lethal, and that the proportion of lethal recombinants lies between 0 and 0.062 (exact binomial confidence interval at the 0.95 level). Tyx was significantly more infectious than Tox at 15 days post inoculation (dpi) (*P* = 0.0026), whereas this difference was no longer statistically significant at 22 dpi (*P* = 0.12). This simply suggests that virus accumulation was below the detection threshold for some of the Tox-inoculated plants at 15 dpi. The infectivity of most (37) of the recombinants did not differ significantly from the infectivity of both parental viruses at the two time points (Figure 2). Three recombinants were significantly more infectious than Tox at 15 dpi. Two recombinants were significantly less infectious than Tyx at 15 dpi, three were significantly less infectious at 22 dpi, and one at both time points. Recombinant 104 was the only clone for which no plant was positive for virus detection at 15 dpi; virus accumulation was probably too low to be detected at this early time point. The most infectious recombinants were those with a high proportion of Tyx sequences. Consistently, there was a significant positive correlation between infectivity and the proportion of the genome derived from Tyx (Figure S3A). As expected, we observed a good linear correlation (*R* ^2^ = 0.618; *P* = 2.2×10^−11^; *t*-test) between the average infectivity of each clone at 15 and 22 dpi across the three experiments conducted at both dates.

To summarize, except for recombinant “104” at 15 dpi, no recombinant had an infectivity significantly different from that of both parents.

### Virus accumulation *in planta*

**Figure 3:**
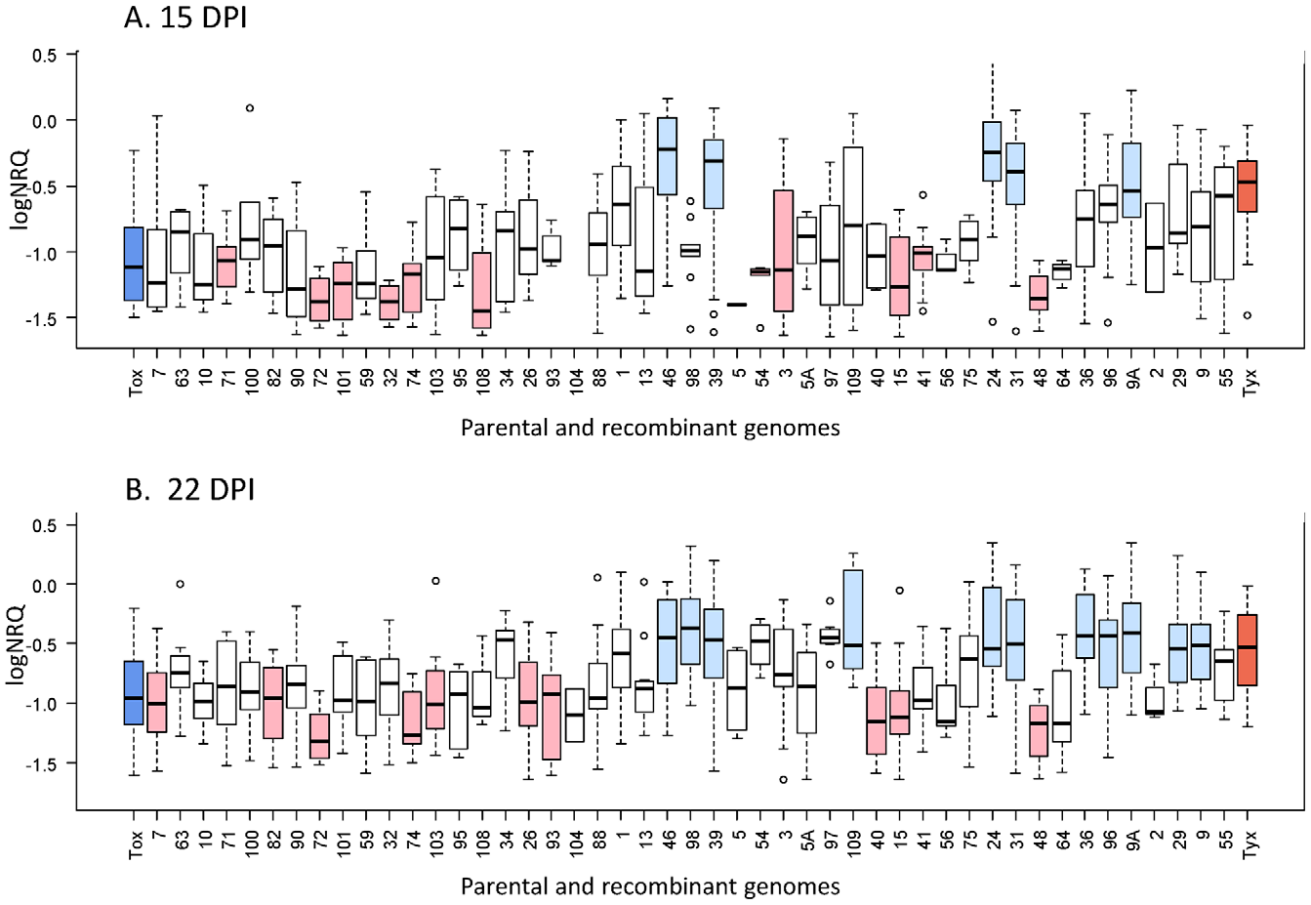
Virus accumulation within infected plants for the 47 recombinants and their two parental genomes. Virus accumulation was measured on plant samples collected at 15 (A) and 22 days (B) post-inoculation. Viral DNA was quantified with real-time PCR. The logarithm of the Calibrated Normalized Relative Quantity (logNRQ) reflects virus accumulation. Within the boxes, the horizontal line indicates the median value (50% quantile), the box itself delimits the 25% and 75% quantiles, and the dashed lines represent the normal range of the values; the points above and/or below correspond to outlying values. The blue box at the left end corresponds to the parental genome Tox and the red box at the right end to the parental genome Tyx. The recombinant genomes are ordered from left to right by increasing nucleotide identity with Tyx genome. White boxes correspond to recombinants that are not significantly different from either parent, light red boxes to recombinants that are significantly less infectious than Tyx, and light blue boxes to recombinants that are significantly more infectious than Tox.

Virus accumulation within inoculated plants was estimated from young leaves at 15 and 22 dpi using quantitative PCR (qPCR). Tyx genomes accumulated to a significantly greater degree than Tox genomes at both 15 and 22 dpi (*P* = 2.3×10^−4^ and *P* = 1.5×10^−4^, respectively). At 15 dpi, virus accumulation of most (30) of the recombinants did not differ significantly from that of both parental genomes; five recombinants accumulated significantly more than Tox, whereas eleven recombinants accumulated significantly less than Tyx (Figure 3A). At 22 dpi, we observed a similar pattern (Figure 3B): virus accumulation in most (25) of the recombinants did not differ significantly from that of both parental genomes, whereas 11 genomes accumulated significantly more than Tox (5 of them also did so at 15 dpi), and 10 genomes accumulated significantly less than Tyx (4 of them also at 15 dpi). The recombinants with a high proportion of genome derived from Tox accumulated within the infected plants at a low level similar to the accumulation of Tox (Figure 3). The recombinants exhibiting the highest virus accumulation were most often the recombinants with a higher proportion of their genome derived from Tyx, the parental virus that accumulated most. Consistently, there was a significant positive correlation between recombinant virus accumulation and the proportion of the genome derived from Tyx (Figure S3B). There were, however exceptions, as for example recombinant 48, in which low virus accumulation was measured despite a high proportion of Tyx. As expected, a good linear correlation (*R* ^2^ = 0.466; *P* = 9.0×10^−8^; *t*-test) was obtained between the average virus accumulation of each clone at 15 dpi and 22 dpi across the three experiments conducted at both time points.

### Distribution of the phenotypic effects of recombination

**Figure 4:**
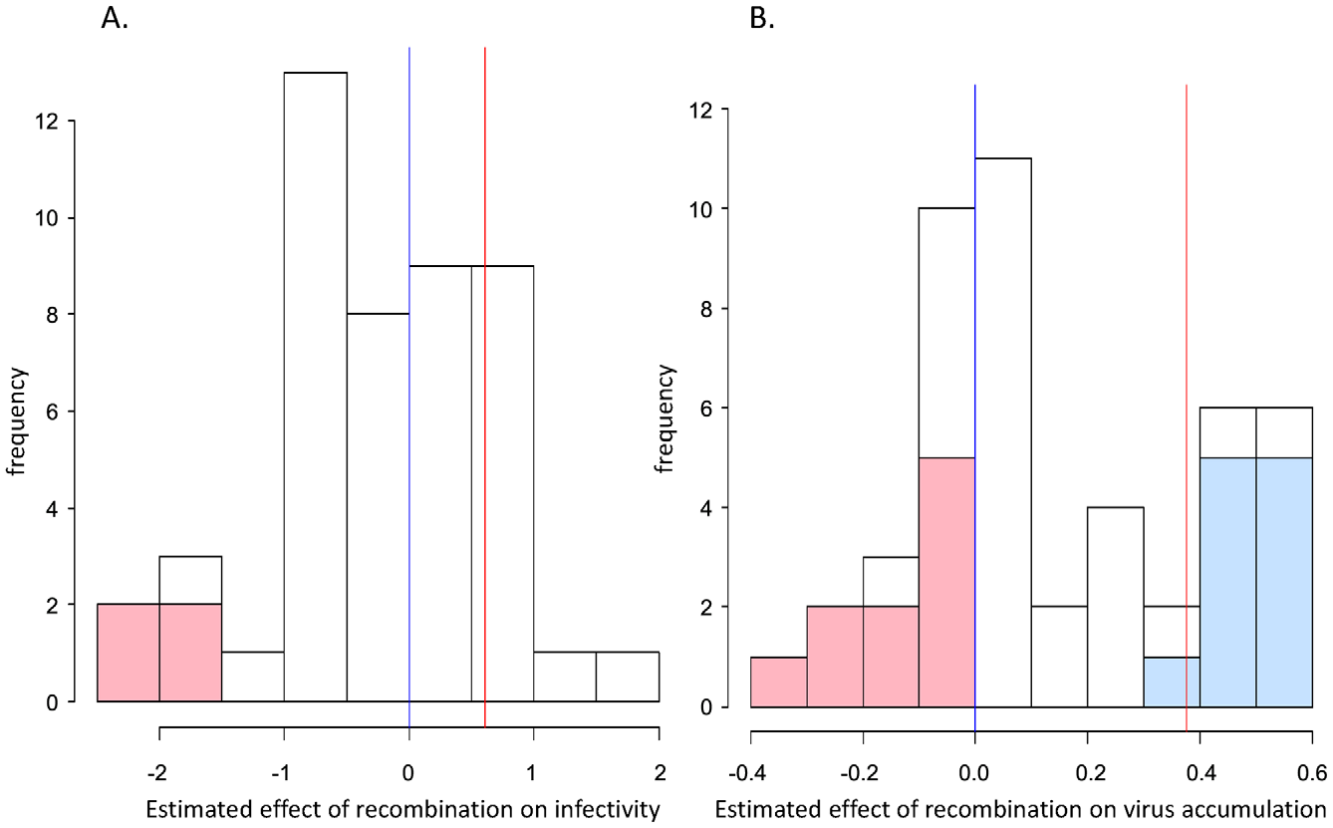
Distribution of the phenotypic effects of random recombination. The effect on the phenotype was assessed through infectivity (A) and virus accumulation (B), at 22 days post inoculation. The estimated effects correspond to the coefficients of the recombinant clones in the corresponding (generalized) linear model. Parental genome coefficients are just superimposed as vertical lines: Tox (blue) and Tyx (red). In each bin, the number of recombinants that differ significantly from Tyx and Tox are represented in light red and light blue, respectively.

The distribution of the effect of recombination on the infectivity and accumulation of virus genomes was derived from the model coefficients corresponding to the 47 recombinant clones (see Materials and Methods). At 22 dpi, the distribution of the effect of recombination on infectivity was unimodal and centred on the Tox parental phenotype (Figure 4A), whereas the distribution of the effect of recombination on virus accumulation was bimodal, with each mode centred on one parental phenotype (Figure 4B).

To summarize, with the exception of recombinant 104, which was not detectable at 15 dpi, no recombinant had a phenotype (infectivity and accumulation) that was significantly different from that of both parents, precisely as expected if recombination had limited effect. Most remarkably, although these two virus species differ genetically by 18%, non-directed recombination did not give rise to any lethal or highly impaired genotype.

## Discussion

An implicit and commonly accepted view of viral recombination is that most recombinant genomes should be deleterious or lethal when the nucleotide identity of the parental sequences drops below 90%. We decided to challenge this view by testing a large set of non-directed recombinants derived from two parental virus species exhibiting only 82% identity. Recombinants were selected randomly from a bank generated by DNA-shuffling—a technology initially applied to random shuffling of individual genes [27] and implemented here for the first time on full-length viral genomes. We chose this methodology as the only technology currently available that allows high-throughput generation of recombinant molecules *in vitro*. The full sequencing of 47 recombinants presented in Figure 1 shows that the distribution of breakpoints and the sizes of exchanged fragments are clearly not random. This deviation from randomization is technically unavoidable as no DNA fragmentation technique presently available can provide completely random fragments. Another step that is impossible to randomize is the hybridization and ligation of these fragments on a parental template to successfully reconstitute full-length “mosaic” genomes. While we alleviated this particular problem by using a mix of both parental genomes as templates, different fragments have different levels of homology (and thus affinity) for these templates and will, or will not, hybridize preferentially to a given region, favoring some breakpoints. Nevertheless, we think that DNA-shuffling remains the best alternative to approximate randomization of DNA fragment exchanges. With this technology, we generated an invaluable genetic resource of hundreds of very diverse recombinants in the absence of the mechanical and selective constraints that occur in nature. For example, the shuffled genome library may be used in the future for the detection of viral molecular determinants of various phenotypic traits by using a quantitative trait loci (QTL) approach.

The view that most recombination events between distantly related (<90% identity) genomes produce deleterious or lethal genotypes has derived from (i) the limited types of recombinants found in the sequence databases, or in co-infected hosts, and (ii) a few experimental estimates of phenotypic traits of selected recombinants. The discrepancy between this view and the results presented here will be discussed in the context of these two types of information.

(i) Recombinant genomes detected either in field populations or in artificially co-infected plants exhibit very specific recombination patterns, leading to the suggestion that other possible patterns were not fit enough to emerge [13], [15], [16]. Likewise, the identification of hot spots of recombination by comparing sequences from databases of virus genomes [17], [18] suggests that recombinants with potential breakpoints in cold spots are maladapted and thus rarely or never isolated. Surprisingly, in our experiment, seven of the eleven recombinants with the highest accumulation *in planta* at 22 dpi were recombined within recombination cold spots previously identified in other members of the genus *Begomovirus* [21]. Moreover, although the sequences located within genes were often reported to be cold spots [13], [28], and particularly when they disrupt protein folding [21], 53 of the 54 generated breakpoints were within coding regions, and 44 of the 47 recombinant genomes encode at least one hybrid protein. The high within-host accumulation of “cold spot” recombinants may thus indicate that their scarcity under natural conditions is not due to a low within-host reproduction capacity, but rather to mechanical constraints hindering their generation during replication. However, it is possible that virus load comparisons in singly infected plants were insufficiently discriminative to distinguish small fitness differences that might be revealed by more comprehensive fitness tests, particularly within-host competition. Unfortunately, competition tests between one recombinant and its parents are not straightforward with this type of viruses. Begomoviruses are notably highly recombinogenic and new recombinants would appear in the co-infected plants with no possibility of distinguishing them from the initially inoculated clones with a qPCR assay.

(ii) The view that most recombination events produce deleterious or lethal genotypes also derives from biological tests of several artificially created recombinants (e.g., 5 in [9], and 17 in [12]). Most of these recombinants were either lethal or accumulated to lower levels than the parental viruses, whose sequence identities were 82% and 75%, respectively. The same conclusion was drawn from recombinants of *Maize streak virus* (MSV, *Geminiviridae*), for which complete genes were swapped between parental genomes with a nucleotide sequence identity of 78-98% [10], [11]. A possible explanation for the viability of all the recombinants obtained in our study, and for the substantial proportion exhibiting a high accumulation level, is that all the recombinants but one (recombinant “2”) contained only parental sequences and lacked any additional changes. This was not the case in a major study in which the phenotype of non-directed homologous recombinants was tested [12], because 13 out of 15 recombinants exhibited additional changes inherent to the RT-PCR-technique with which they were produced. Another hypothesis explaining the absence of lethal or highly deleterious effects of recombination in our system may be that the two parental viruses were isolated from the same host (tomato), whereas in earlier studies, parental viruses were often isolated from different host species [10], [11], [12]. This hypothesis would suggest that the relatively low accumulation of some recombinants in these earlier studies resulted from a selective cost induced by host-specific mutations [29], [30] rather than from the effect of recombination *per se*.

A very interesting study compared β-lactamase variants created by DNA-shuffling of different pre-existing genes [31], with variants created by non-directed mutations, all containing the same number of amino acid substitutions [6]. The variants created by recombination retained function with a significantly higher probability than those generated by random mutagenesis. The authors explained the less destructive nature of recombination, at least in part, by the fact that amino acids exchanged by recombination have already proven compatible with the homologous structure from which they originate, whereas substitutions created by mutation have not been pre-selected in any context. An interesting comparison can be drawn between the results obtained here on the phenotypic effects of recombination in viruses, and results from similar approaches carried out earlier on the phenotypic effects of random mutation [7], [8]. While we conclude that lethal or largely deleterious effects of recombination can be very rare even for parental genomes differing by up to 20%, non-directed mutation was reported to induce up to 40% lethal genomes. Interestingly, this comparison suggests that a phenomenon observed on one isolated gene (β-lactamase) may also be valid for whole viral genomes. The recombinant viral genome library generated here could be used in the future to test this fascinating prediction, by creating and comparing a set of randomly mutated genomes of Tyx and/or Tox that would exhibit the same number of amino acid substitutions as the recombinant genomes described above.

Data generated in this paper not only address fundamental questions regarding recombination, but are also relevant to the economically important question of emerging diseases. In our specific example, although the 2 parental viruses (TYLCV, ToLCYTV) originate from distinct hemispheres, they were recently brought into close proximity in the South West Indian Ocean region [32], [33]. The probability of their encounter in the same island, and thus the probability of generating natural recombinants, is now very high. The risk of emergence of TYLCV/ToLCYTV recombinants with altered phenotypes can be further evaluated using the recombinants described here, and also by searching for other recombinants in the available Tyx/Tox-shuffled bank. The economic importance of the emergence of TYLCV/ToLCYTV recombinants can be further assessed by testing the virulence (aggressiveness) and vector transmission (by the whitefly *Bemisia tabaci*) of the shuffled clones. Finally, estimation of all these biological parameters may be used in the future to test the trade-off hypothesis related to evolution of virulence, which predicts a positive correlation between virus accumulation, virulence and transmission rate [34].

## Materials and Methods

This work did not involve any human participant and include any animal work that would require an ethics statement.

### Parental viral genomes

The parental viruses are members of the genus *Begomovirus*, family *Geminiviridae*. One of the parental genomes, the "Mild" strain of *Tomato yellow leaf curl virus* (TYLCV-Mld, accession no. AJ865337), was isolated from tomato plants (*Solanum lycopersicum*) collected in Réunion island in 2002 [32]. The other parental genome, *Tomato leaf curl Mayotte virus* (ToLCYTV-[Dem] accession no. AJ865341) was isolated from tomato collected in Dembeni (Mayotte) in 2003 [32]. Both isolates were originally cloned individually into pGEMT. In order to render these clones directly infectious, we created agroinfectious single-unit length viral genomes for both parental clones as described previously [35], except that the engineered unique cloning site was *Xho*I instead of *Not* I, and the binary plasmid vector was pCambia0380. Briefly, a unique *Xho*I site was generated in both pGEMT clones (Figure S4). The conserved stem loop containing the viral origin of replication and the unique *Xho*I site was synthesized with 2 complementary oligonucleotides and inserted into the *Sma*I site within the multicloning site of the pCambia0380 plasmid (Figure S4). The *Xho*I-modified parental clones were inserted into the *Xho*I site of the modified pCambia0380 so that the repeated stem loops were in the sense orientation to allow replicational release. These cloned parental genomes—called Tyx (for TYLCV-Mld) and Tox (for ToLCYTV-[Dem])—were checked for infectivity and subsequently used for L-DNA-shuffling.

### L-DNA-shuffling

The two parental genomes Tyx and Tox were released from pCambia0380 using two unique restriction sites (*Sac*II and *Bgl*II) located in the flanking sequences in the plasmid. Recombinant viral genomes were produced using the patented L-DNA-shuffling technology (European Patent 1104457, US Patent 6951719) developed by Proteus (Nîmes, France). We chose this particular approach because, in contrast to other DNA-shuffling technologies, L-DNA-shuffling does not include any PCR amplification step that could potentially induce additional mutations in the generated recombinants. Since this technology has been used successfully to shuffle long genes with high or low nucleotide identity, it seemed particularly convenient for shuffling small complete viral genomes consisting of genes and intergenic regions with varying levels of nucleotide identity.

L-DNA-shuffling is a four-step procedure: (i) fragmentation of the parental genomes TYLCV and ToLCYTV using a non-specific endonuclease; (ii) denaturation; (iii) hybridisation of the fragments onto templates—in our case a mix of the full-length parental genome sequences at a 1∶1 ratio; (iv) ligation of adjacent fragments after elimination of overlapping sequence ends. Steps (ii) to (iv) were cycled until a large amount of full-length “mosaic” (recombinant) sequences were obtained.

In step (i), the size of the genome fragments generated depends on the duration of the endonuclease incubation step. To increase the size diversity of the fragments exchanged in the final recombined viral genomes, two distinct endonuclease incubations were performed: long and short incubations were used to generate fragments with sizes following a normal distribution centred on lengths of ∼50 nt and ∼500 nt, respectively. The endonuclease products of the long and short incubations were mixed before proceeding to steps (ii), (iii) and (iv). A sample of the final shuffled products was digested with *Sac*II and *Bgl*II (NEB) and inserted into the plasmid pCambia0380 at the corresponding restriction sites to generate a bank of mosaic recombinant full-length genomes. An aliquot of the ligation product was introduced into *Escherichia coli* MC1061 DE3 via electroporation.

Clones were picked at random from the hundreds of bacterial colonies obtained, and their plasmid DNA was extracted and fully sequenced. Around 30% of these plasmids contained one or the other of the parental viral sequences (Tyx or Tox), whereas 70% contained recombinant viral genomes. This search and sequencing of viral genomes was continued until 47 distinct recombinants were identified, as this was the maximum number that could be tested simultaneously in our containment chamber with both parents and a negative control. A total of 48 recombinant genomes had to be sequenced because two of them were identical.

### Plant inoculation and growth conditions

The pCambia0380 plasmids containing the recombinant genomes were purified from *E. coli* and introduced into *Agrobacterium tumefaciens* C58 MP90 by electroporation. Transformed *A. tumefaciens* were cultivated overnight at 28°C in a liquid LB medium containing kanamycin and gentamycin. As soon as the cultures reached an optical density of between 2 and 5, the bacterial suspension was concentrated 10 times by centrifugation and resuspended in sterile water for agroinfiltration as described below.

Tomato plants of the susceptible cultivar Monalbo (INRA) were grown in containment growth chambers under 14 h light at 26°C, and 10 h dark at 24°C. Seeds were initially grown in batches and were transplanted to individual pots after 7 days. During development, plants were irrigated with 15∶10∶30 NPK+ oligoelements.

Tomato plants at the one-leaf stage were agroinfiltrated on each cotyledon with a syringe. Within the same growth chamber, 47 recombinant clones, the two parental clones Tyx and Tox, and a clone containing an empty pCambia0380 plasmid as a negative control, were inoculated on the same day onto eight plantlets each. The inoculated plants were randomised completely and grown until 22 dpi. The whole experiment was repeated four times, each repeat representing an independent test. In the first test, plants were sampled at 22 dpi, corresponding approximately to the delay required to reach the maximum virus accumulation level of the parental strain TYLCV-Mild [35]. In the three following tests, plants were also collected at 15 dpi, a time point at which viral clones may be distinguished by their speed in reaching the maximum virus accumulation level [35].

### Sampling of plant material and DNA extraction

TYLCV has been shown to accumulate mainly at the top of infected tomato plants [36], and the four youngest leaves of a plant infected with a related tomato begomovirus (*Tomato yellow leaf curl Sardinia virus*) were shown using real-time PCR to accumulate similar amounts of viral DNA [37]. We thus considered that a relevant and reliable estimation of the accumulation of both Tyx and Tox within each plant could be obtained from measurement of the viral DNA concentration in one of the four youngest leaves developing below the apex. More specifically, from each infected plant, we collected a 5 mm-diameter leaf disk from the youngest leaf for which five leaflets were visible. Total DNA from each leaf disk was extracted with the QuickExtract kit from Epicentre Biotechnologies (Madison, WI, USA) according to the manufacturer's recommendations.

### Quantification of viral DNA accumulation in plants using real-time PCR

Two microlitres of a 1/10 dilution of the total DNA extract was added to 8 µl of a qPCR mix (LightCycler 480 SYBR Green I Master, Roche). The amplification reactions were run in 384-well optical plates in the LightCycler 480 (Roche). Primers were targeted to regions conserved in both parental genomes to ensure similar amplification from all recombinant genomes; the respective sequences of forward and reverse primers were: Ty2164+ (CTAAGAGCCTCTGACTTACTGC) and Ty2339- (AACATTCAGGGAGCTAAATCCAG). To standardize all measurements of viral DNA accumulation, we quantified the amount of plant DNA in each extracted sample by targeting the actin2 gene in parallel real-time PCR amplifications. The primers used were Act1+ (CCCRGAGGTHCTCTTCCARC) and Act148- (TMCGRTCAGCAATACCAGGG).

To estimate viral accumulation in each analysed sample, we used the program LinRegPCR, which is based on the procedure described by Ruijter et al. (2009), and the Pfafll (2001) quantification model. The procedure used by Ruijter et al. (2009) assesses PCR efficiency in each well. Coupling this approach to Pfaffl's relative quantification model has the advantage of minimizing the propagation of errors in (i) setting the fluorescence threshold, (ii) determining the Ct value (fractional cycle number required to reach the fluorescence threshold), (iii) estimating the baseline efficiency, and (iv) accounting for intra- and inter-test variation. The variation between samples due to the high quantity of qPCR runs required for this experiment was minimized by an inter-plate calibrator that was used in each plate for both viral and actin2 DNA quantification. The inter-plate calibrator was prepared from a single extraction of a symptomatic Tyx-infected plant. The highly concentrated extract was split into multiple aliquots conserved at −20°C, each aliquot being used for one qPCR run only. After checking key quality control points—technical replicates, negative controls and positive controls—we calculated a Calibrated Normalized Relative Quantification (CNRQ) value for all samples according to equation 3 in [38]:
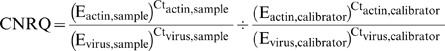



“E_actin, sample_” and “E_virus, sample_” are the PCR efficiencies calculated for each well containing a plant extract sample, for actin2 and virus DNA quantification, respectively. Similarly, “E_actin, calibrator_” and “E_virus, calibrator_” are the PCR efficiencies calculated for each calibrator well, for actin2 and virus DNA quantification, respectively. CNRQ values reflect virus accumulation in each individual plant. A plant was defined as infected when its Log(CNRQ) value was above the 95%-quantile of the distribution of Log(CNRQ) values corresponding to 92 samples from mock-inoculated plants.

### Statistical analysis

At each of the two sampling time points (15 dpi and 22 dpi), infectivity was modeled as the result of a binomial experiment where the number of inoculated plants that became infected or non-infected depends on the effect of the clone (2 parental genomes and 47 recombinants), adjusted for the effect of the experiment (the experiment was replicated 4 times). The clone×experiment interaction was not included because it would have resulted in an overparameterized model. The analysis of deviance for the corresponding generalized linear model (GLM) indicated that both the experiment and the clone had a very significant effect at 15 dpi (chi-square test; *P* = 6.8×10^−12^ and *P* = 1.1×10^−18^, respectively) and at 22 dpi (chi-square test; *P* = 2.6×10^−10^ and *P* = 5.1×10^−16^, respectively). The model residuals did not show any pathological behavior.

At each of the two sampling time points (15 dpi and 22 dpi), virus accumulation in infected plants [Log(CNRQ)] was modeled as a linear model where the effect of the clone was adjusted for the effect of the experiment. The clone×experiment interaction was not significant (F-test; *P* = 0.61 at 15 dpi and *P* = 0.72 at 22 dpi). The analysis of variance for the corresponding linear model (LM) indicated a significant experiment effect and a very significant clone effect at 15 dpi (F-test; *P* = 0.023 and *P* = 4.5×10^−16^, respectively) and at 22 dpi (F-test; *P* = 1.5×10^−3^ and *P*<2.2×10^−16^, respectively). The model residuals did not show any pathological behavior.

In each model, taking Tox as the reference clone, the coefficients corresponding to the clone effects (adjusted for the experiment effect) were used for: (i) testing whether the effect of the two parental genomes was significantly different (z-test for the GLM, *t*-test for the LM), (ii) identifying which recombinant clones had an effect that differed significantly (at the 0.05 level) from the effect of the Tyx (resp. Tox) parent (using *z*-tests for the GLM and *t*-tests for the LM, followed by the Holm multiple-testing correction), (iii) constructing the distribution of the effects of all the recombinant clones. All statistical analyses were performed using R version 2.8.1 [39].

## Acknowledgments

We are thankful to Yannis Michalakis for discussing experimental design. We thank Jean-Luc Macia and Christophe Michel for their excellent technical assistance in growing and sampling the plants. We are grateful to André Moretti of INRA (Avignon, France) for the production and kind supply of the tomato seeds.

## Supporting Information

Figure S1 Location and frequency of breakpoints detected along the genomes of the 47 randomly selected recombinants. (A) Location of nucleotide positions where the two parental genomes differ. The nucleotide numbering of the alignment of the parental genomes is indicated below on the x-axis, with position 0 corresponding to the *Xho*I cloning site at the 3′ side of the conserved stem-loop. Each thin vertical line indicates one nucleotide difference between Tyx and Tox. (B) Location and frequency of breakpoints found in the 47 analyzed recombinants. Each adjacent discriminating position between Tyx and Tox (shown in A) was used to delimit a region within which fragments originating from the different parental genomes were ligated during the L-DNA-shuffling procedure. The y-axis represents the frequency with which a breakpoint was detected within one of these particular delimited regions. The positions of the 6 ORFs encoded by the parental genomes are indicated below the graph. The red and blue horizontal arrows represent recombination hot and cold spots, respectively, as described in the literature from the sequences of geminivirus genomes available in databanks [21]; the red and blue numbers indicate the number of distinct breakpoints that were detected within these regions in the 47 recombinants presented in Figure 1.
(TIF)

Figure S2 Distribution of the size of the recombined genomic fragments generated by L-DNA-shuffling technology detected in the 47 randomly selected recombinants.
(TIF)

Figure S3 Correlation at 22 days post inoculation between the proportion of Tyx genome and (A) infectivity and (B) virus accumulation. The circles represent the 47 recombinants and the two parental genomes; a smooth trend line is added in red. The linear regression line is represented in black, with *R* ^2^ = 0.102 (*P* = 0.025; t-test) for infectivity and *R* ^2^ = 0.211 (*P* = 8.9×10^−4^; t-test) for virus accumulation.
(TIF)

Figure S4 Creation of full-length infectious parental clones, Tyx and Tox, in the binary vector pCambia0380 according to [35]. (A) Site-directed mutagenesis at the end of the stem-loop to generate an *Xho*I site in TYLCV and ToLCYTV. A second site-directed mutagenesis modified a “T” of the stem loop of TYLC to “C”, eliminating a mismatch within the stem that is present only in the original sequence of TYLCV. Nucleotides mutated in the parental sequences (top and bottom lines) to create a common stem loop and a cloning site (middle line) are indicated in lower case. (B) Oligonucleotide sequence used to insert the common stem loop shown in A (middle line) at the *Sma*I site of the multiple cloning site of pCambia0380. The solid horizontal lines represent the inverted repeats that constitute the stem of the stem-loop, and the dotted lines represent restriction sites. The stars indicate the origin of the rolling circle replication of the viral genomes.
(TIF)
