# Supplementary figures and images for: Distribution of the Phenotypic Effects of Random Homologous Recombination between Two Virus Species
Source: PLoS Pathog. 2011 May 5;7(5):e1002028. doi: 10.1371/journal.ppat.1002028 (PMC3088723; doi:10.1371/journal.ppat.1002028)

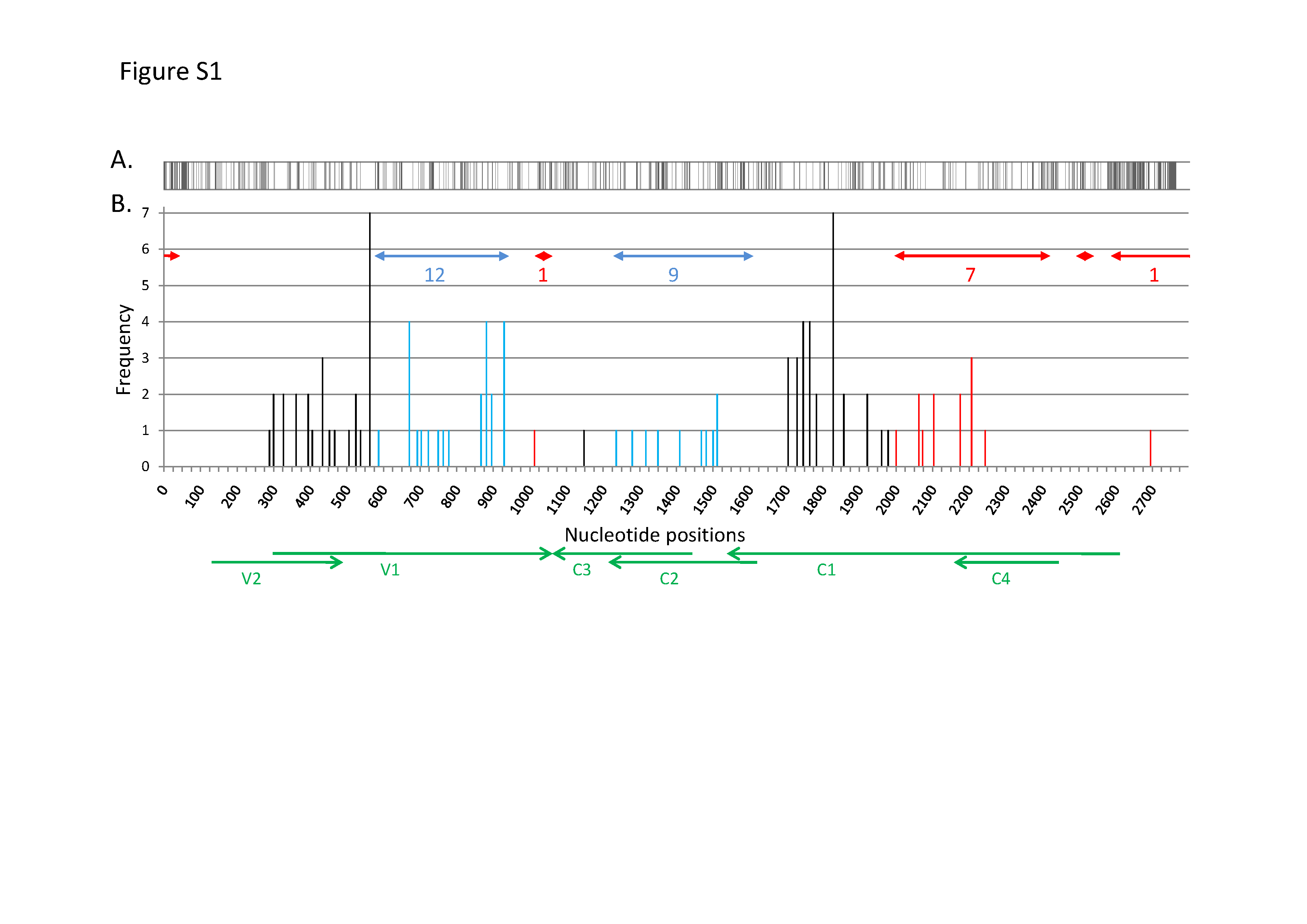

Supplement: Figure S1 — Location and frequency of breakpoints detected along the genomes of the 47 randomly selected recombinants. (A) Location of nucleotide positions where the two parental genomes differ. The nucleotide numbering of the alignment of the parental genomes is indicated below on the x-axis, with position 0 corresponding to the XhoI cloning site at the 3′ side of the conserved stem-loop. Each thin vertical line indicates one nucleotide difference between Tyx and Tox. (B) Location and frequency of breakpoints found in the 47 analyzed recombinants. Each adjacent discriminating position between Tyx and Tox (shown in A) was used to delimit a region within which fragments originating from the different parental genomes were ligated during the L-DNA-shuffling procedure. The y-axis represents the frequency with which a breakpoint was detected within one of these particular delimited regions. The positions of the 6 ORFs encoded by the parental genomes are indicated below the graph. The red and blue horizontal arrows represent recombination hot and cold spots, respectively, as described in the literature from the sequences of geminivirus genomes available in databanks [21]; the red and blue numbers indicate the number of distinct breakpoints that were detected within these regions in the 47 recombinants presented in Figure 1. (TIF) [file ppat.1002028.s001.tif]

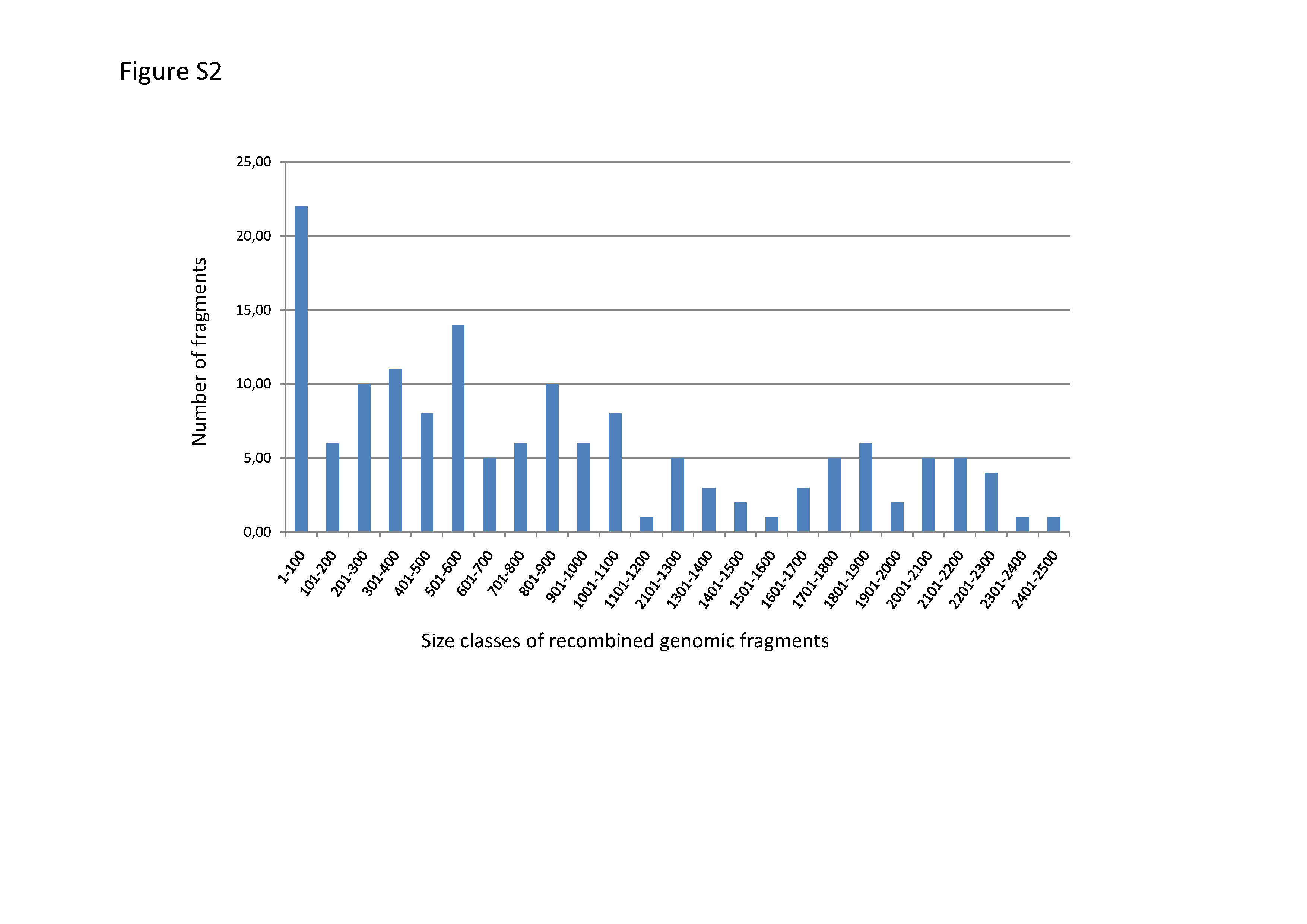

Supplement: Figure S2 — Distribution of the size of the recombined genomic fragments generated by L-DNA-shuffling technology detected in the 47 randomly selected recombinants. (TIF) [file ppat.1002028.s002.tif]

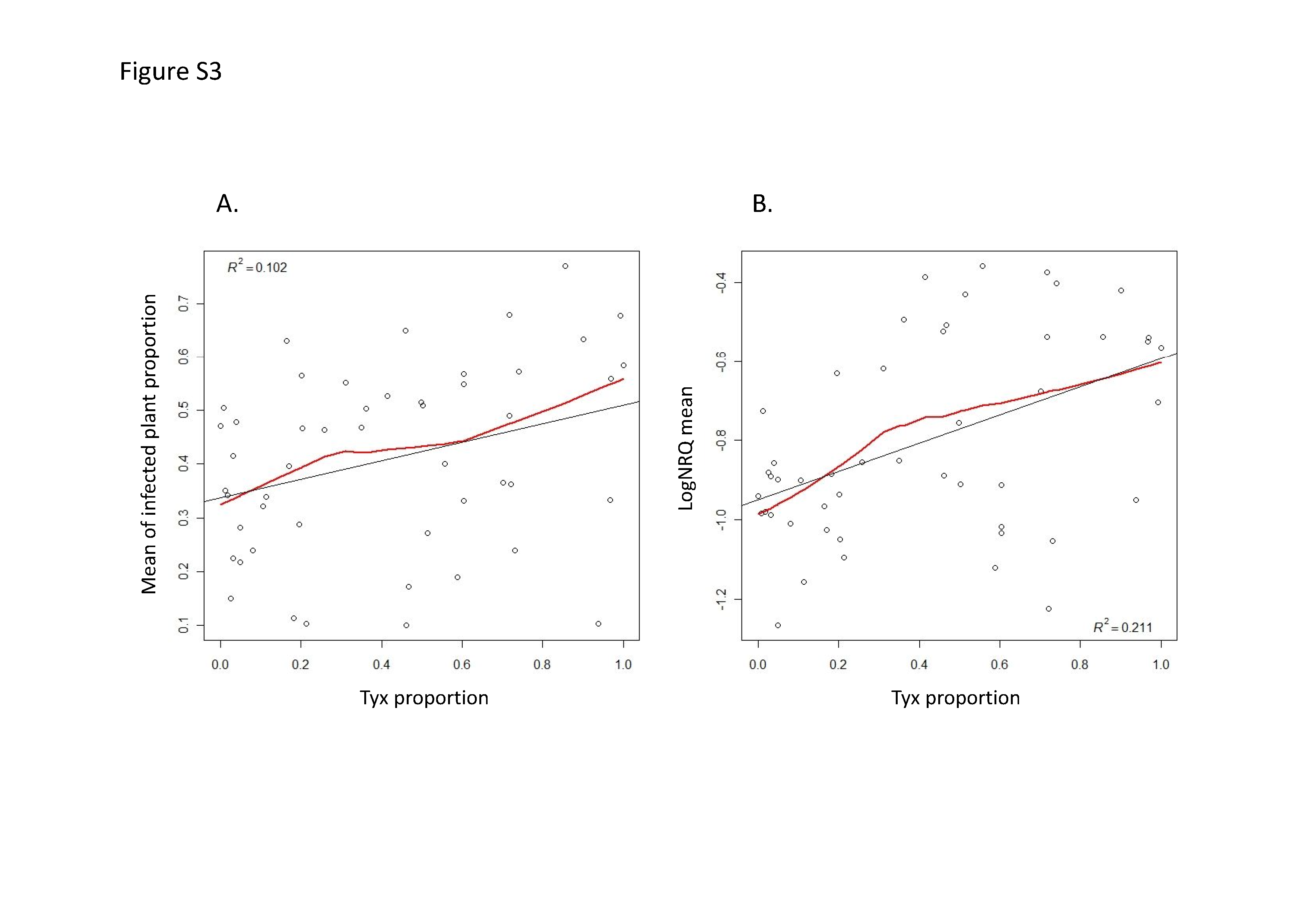

Supplement: Figure S3 — Correlation at 22 days post inoculation between the proportion of Tyx genome and (A) infectivity and (B) virus accumulation. The circles represent the 47 recombinants and the two parental genomes; a smooth trend line is added in red. The linear regression line is represented in black, with R 2 = 0.102 (P = 0.025; t-test) for infectivity and R 2 = 0.211 (P = 8.9×10^−4^; t-test) for virus accumulation. (TIF) [file ppat.1002028.s003.tif]

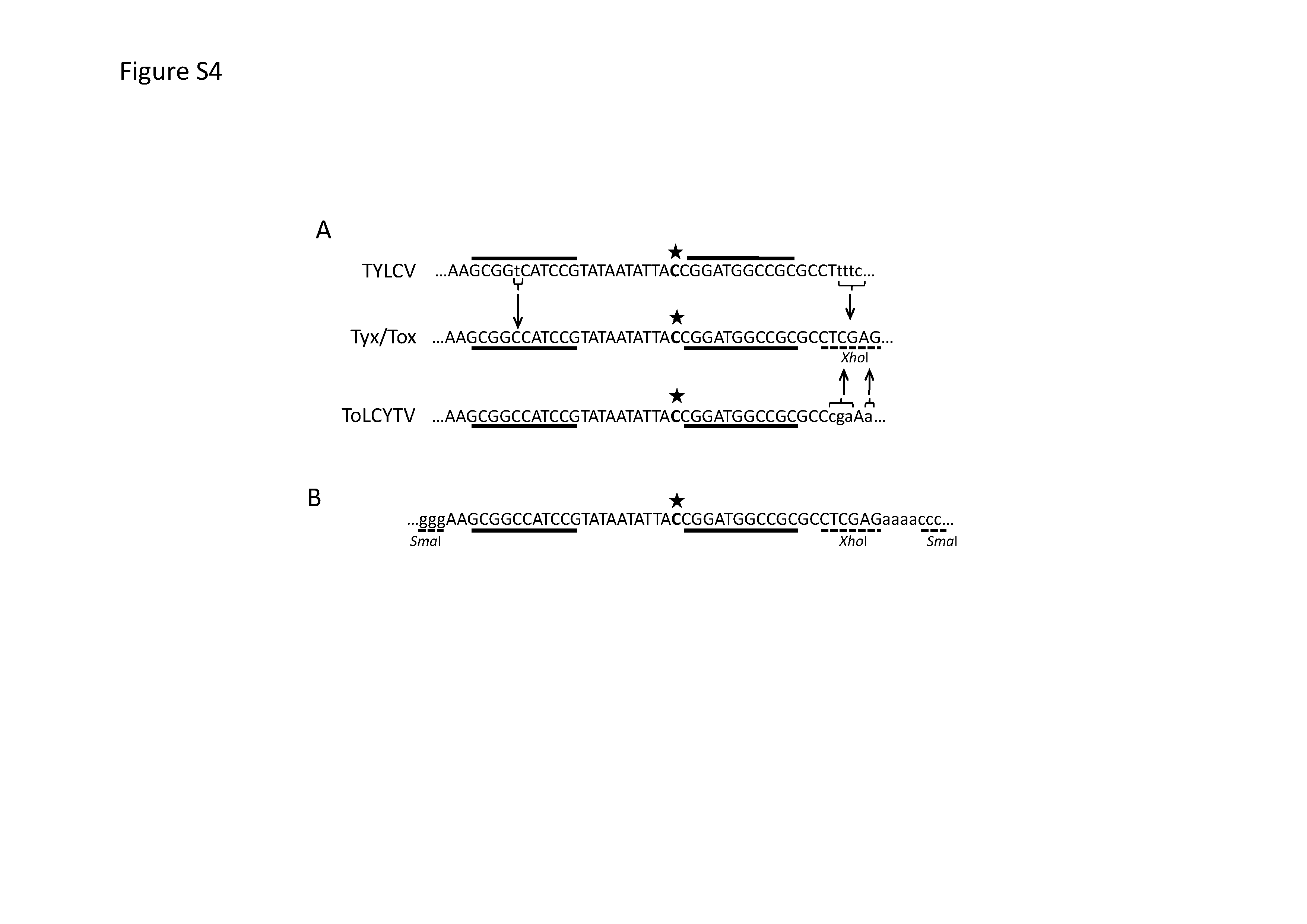

Supplement: Figure S4 — Creation of full-length infectious parental clones, Tyx and Tox, in the binary vector pCambia0380 according to [35]. (A) Site-directed mutagenesis at the end of the stem-loop to generate an XhoI site in TYLCV and ToLCYTV. A second site-directed mutagenesis modified a “T” of the stem loop of TYLC to “C”, eliminating a mismatch within the stem that is present only in the original sequence of TYLCV. Nucleotides mutated in the parental sequences (top and bottom lines) to create a common stem loop and a cloning site (middle line) are indicated in lower case. (B) Oligonucleotide sequence used to insert the common stem loop shown in A (middle line) at the SmaI site of the multiple cloning site of pCambia0380. The solid horizontal lines represent the inverted repeats that constitute the stem of the stem-loop, and the dotted lines represent restriction sites. The stars indicate the origin of the rolling circle replication of the viral genomes. (TIF) [file ppat.1002028.s004.tif]
